# Free-Living Species of Carnivorous Mammals in Poland: Red Fox, Beech Marten, and Raccoon as a Potential Reservoir of *Salmonella*, *Yersinia*, *Listeria* spp. and Coagulase-Positive *Staphylococcus*

**DOI:** 10.1371/journal.pone.0155533

**Published:** 2016-05-12

**Authors:** Aneta Nowakiewicz, Przemysław Zięba, Grażyna Ziółkowska, Sebastian Gnat, Marta Muszyńska, Krzysztof Tomczuk, Barbara Majer Dziedzic, Łukasz Ulbrych, Aleksandra Trościańczyk

**Affiliations:** 1 Sub-Department of Veterinary Microbiology, Institute of Biological Bases of Animal Diseases, Faculty of Veterinary Medicine University of Life Sciences, Lublin, Poland; 2 State Veterinary Laboratory, Lublin, Poland; 3 Sub-Department of Parasitology and Invasive Diseases, Institute of Biological Bases of Animal Diseases, Faculty of Veterinary Medicine, University of Life Sciences, Lublin, Poland; 4 Warta Mouth National Park, Górzyca, Poland; US Geological Survey, UNITED STATES

## Abstract

The objective of the study was to examine a population of free-living carnivorous mammals most commonly found in Poland (red fox, beech marten, and raccoon) for the occurrence of bacteria that are potentially pathogenic for humans and other animal species and to determine their virulence potential (the presence of selected virulence genes). From the total pool of isolates obtained (n = 328), we selected 90 belonging to species that pose the greatest potential threat to human health: *Salmonella* spp. (n = 19; 4.51%), *Yersinia enterocolitica* (n = 10; 2.37%), *Listeria monocytogenes* and *L*. *ivanovii* (n = 21), and *Staphylococcus aureus* (n = 40; 9.5%). The *Salmonella* spp. isolates represented three different subspecies; *S*. *enterica* subsp. *enterica* accounted for a significant proportion (15/19), and most of the serotypes isolated (*S*. Typhimurium, *S*. Infantis, *S*. Newport and *S*. Enteritidis) were among the 10 non-typhoidal *Salmonella* serotypes that are most often responsible for infections in Europe, including Poland. *Y*. *enterococlitica* was detected in the smallest percentage of animals, but 60% of strains among the isolates tested possessed the *ail* gene, which is responsible for attachment and invasion. Potentially pathogenic *Listeria* species were isolated from approx. 5% of the animals. The presence of all tested virulence genes was shown in 35% of *L*. *monocytogenes* strains, while in the case of the other strains, the genes occurred in varying numbers and configurations. The presence of the *inl*A, *inl*C, *hly*A, and *iap* genes was noted in all strains, whereas the genes encoding PI-PLC, actin, and internalin Imo2821 were present in varying percentages (from 80% to 55%). *S*. *aureus* was obtained from 40 individuals. Most isolates possessed the *hla*, *hld* (95% for each), and *hlb* (32.5%) genes encoding hemolysins as well as the gene encoding leukotoxin lukED (70%). In a similar percentage of strains (77.5%), the presence of at least one gene encoding enterotoxin was found, with 12.5% exhibiting the presence of *egc-*like variants. In two animals, we also noted the gene encoding the TSST-1 toxin. The results of the study showed that free-living animals may be a significant reservoir of bacteria that are potentially pathogenic for humans. The results of the statistical analysis revealed that, among the animals species studied, the red fox constitutes the most important source of infections.

## Introduction

Free-living animals have always been a significant source of infectious diseases transmitted to humans. Zoonoses whose reservoir is this group of animals are currently a serious problem [1], particularly as an increasing number of pathogens appear to be of animal origin. Free-living carnivorous animals are a permanent biocoenotic element that is highly diverse in terms of adaptation to the environment; in general, they have outstanding migratory potential associated with an active search for food. These predators not only live in environments without people, but also appear in areas populated by humans, particularly in the winter or spring when there is considerably less available food [2]. Species such as the red fox, beech marten, or raccoon constitute a relatively large percentage of the population of free-living carnivorous animals in Poland. The red fox *Vulpes vulpes* (Linnaeus, 1758) is a common species all over Europe and its population in Poland is estimated at 200,000–250,000 individuals [3]. Its high level of synanthropization, manifested as gradual colonization of suburban and urban areas, may significantly increase the possibility of contact with humans and thus the probability of transfer of pathogenic agents onto both humans and other domesticated animals [4,5]. The beech marten *Martes foina* (Erxleben, 1777) inhabits most regions of Europe and is found all over Poland, where its population is difficult to estimate, but it is probably approx. 100,000. The species is closely associated with environments populated by humans [6]. Both the red fox and the beech marten are common in Europe. In Poland, they have a status of game species with a closed period [6]. The raccoon *Procyon lotor* (Linnaeus, 1758) is the most recently recorded alien species in Poland. It originally lived in North America. In 1934, raccoons were introduced in Germany and the first record of the presence of the raccoon in Poland dates back to 1970. Since then, the raccoon population has grown fairly rapidly, particularly in northern and western Poland. Raccoons are now a permanent component of both the natural environment [7] and urban areas (large and smaller cities) in this part of the country [8], but research conducted in 2005–2009 also showed isolated cases of the presence of individuals of this species in other regions of Poland. The most serious problem is their invasion into protected natural areas, which is one of the reasons why raccoons are game animals that can be hunted year round with no closed season.

A characteristic trait of these species of free-living animals is a broad-spectrum diet, including all vertebrates, numerous invertebrates, and carrion. Carnivorous animals at the top of the food chain can therefore become carriers of a broad spectrum of microbes that are potentially pathogenic for humans. The clear tendency towards synanthropization in these species suggests the possibility of direct contact with the living environment of humans, which may increase the likelihood of transmission and spread of microbes, including those posing a threat to public health [5,9]

Analysis of these species in terms of carriage of zoonotic diseases in Poland primarily focuses on detection of the rabies virus. Monitoring for this virus, particularly in foxes, which are its main reservoir in Europe [10], is legally sanctioned. The importance of these animals as a potential source of parasitoses of animal origin, such as *Echinococcus multilocularis* in foxes [1, 11] and *Baylisascaris procyonis* in raccoons [12] has also been confirmed.

Data on the occurrence and isolation of other microorganisms in wildlife animals are fragmentary and either concern single cases of isolation or occurrence in other free-living animals species, e.g. isolation of *Mycobacterium bovis* from wild boar [13], prevalence of *Salmonella* in free-living birds [14], or detection of the HEV virus in wild boar [15].

The objective of the study was to examine red fox, beech marten, and raccoon populations for the occurrence of bacteria that may be potentially pathogenic agents for humans and for other animal species and to determine their virulence potential.

## Material and Methods

### Animals used in the study and sample collection

Samples in the form of rectal swabs were collected from May 2011 to April 2015 in western Poland (52.593333, 14.748333) and in southern and south-eastern Poland (51.216667, 22.9) from the following free-living species: the red fox *Vulpes vulpes* (n = 286), beech marten *Martes foina* (n = 65), and raccoon *Procyon lotor* (n = 70). The samples were taken from foxes that were killed under a program monitoring the effectiveness of rabies vaccination [16] and from martens supplied to the laboratory due to suspected infection of rabies, according to regulations on combating infectious diseases in animals. The animals were not sacrificed solely for the sake of the sampling for the research conducted in this study. Since cases of rabies in animals are still detected in Poland, in the case of suspected symptoms of rabies, free-living animals, including foxes and martens, shall be subject to diagnostics observation by qualified veterinary staff and, in the case of death during the observation, these animals are transferred for rabies testing to an official laboratory. In addition, in the case of foxes, there is mandatory assessment of the effectiveness of the vaccination against rabies and, therefore, every year a certain number of animals must be shot by hunters and delivered to an official laboratory for appropriate tests [16]. Because the material was obtained from dead animals analyzed at the State Veterinary Laboratory on the basis of the Act on protection of animal health and combating infectious diseases in animals (Dz.U. [Journal of Laws] 2004 no. 69 item 625), no approval of the ethics commission was required.

Bacteriological examination of the samples was performed only after virological tests had been completed with negative results. Until that time, they were stored at -80°C for maximum 30 days.

Samples from the raccoons were taken from individuals captured in the environment (Warta Mouth National Park) in restraining traps and then the animals were subjected to euthanasia by qualified veterinary staff (prior pharmacological premedication) under the project LIFE+ No: LIFE09 NAT/PL/000263 entitled ‘Protection of water and marsh birds in five National Parks–reconstructing habitats and curbing the influence of invasive species’ [17]. As an invasive species in Poland, the raccoon may pose a threat to native species in some specific areas (e.g. national parks or nature reserves) and a specified part of the invasive animal population need to be caught and euthanized for biodiversity conservation. The material was collected in sterile conditions at the State Veterinary Laboratory immediately after the dead animals were delivered, and bacteriological analysis was performed within 24 h.

The direction of the research was chosen on the basis of the available literature on potential bacterial threats to human health in the environment [18, 19, 20].

The samples were initially incubated in 2 mL of buffered peptone water (24 h at 37°C). *Salmonella* were isolated according to the Polish standard [21]. Five typical colonies were selected from each culture for further identification. Bacterial species were confirmed using a commercial test (ENTEROtest 24N, Erba Lachema, Brno, Czech Republic) and serotyping was performed using commercial sera for O and H antigens (SIFIN, Berlin, Germany), according to the Kauffman-White classification [22].

*Listeria* spp. were isolated according to the Polish standard [23]. One typical colony was selected from each culture for further identification. Species identification was based on PCR-restriction fragment length polymorphism according to Paillard et al. [24]. All isolates of *L*. *monocytogenes* were tested by PCR for the presence of the following virulence genes: *inl*A, *inl*C, *inl*J (internalins), *iap* (protein p60), *hly*A (listeriolysin O—LLO), *plc*A (phosphatidylinositol-phospholipase C–PI-PLC), *act*A (surface protein ActA), and *prf*A (virulence regulator PrfA). PCR primers and conditions followed those described in previously established protocols [25].

The *Yersinia* spp. isolation procedure consisted in cold incubation at 5°C for 7 d in Tripticase Soy Broth (Biocorp, Warsaw, Poland) followed by inoculation on Yersinia CIN Lab Agar (Biocorp, Warsaw, Poland) at 25°C for 24 h. Colonies that were morphologically similar to *Yersinia* spp. were subcultured for biochemical examination with the commercial test ENTEROtest 24N (Erba Lachema, Brno, Czech Republic). Genotypic species identification (based on analysis of the 16S rRNA gene) and the pathogenicity of *Y*. *enterocolitica* strains (the presence of the *ail* gene) were determined by duplex PCR according to Wannet et al. [26].

*Staphylococcus* spp. were isolated according to the Polish standard [27]. One typical colony was selected from each culture for further identification. Species identification of isolates was carried out with the commercial kit STAPHYtest 24 (Erba Lachema, Brno, Czech Republic). Species identification of coagulase-positive isolates was confirmed according to Sasaki et al. [28]. In addition, tests were performed for the presence of coagulase (using rabbit coagulase plasma, Biocorp, Warsaw, Poland) and for the presence of DNAse (DNA-se test Lab Agar Biocorp, Warsaw Poland). All *S*. *aureus* isolates were tested by PCR for the presence of genes coding for staphylococcal enterotoxins (*sea*, *seb*, *sec*, *sed*, *see*, *seg*, *seh*, *sei*, *sej*, *sen*, *seo*, and *sem*) [29], leukocidin genes (*luk*S/F-PV and *luk*M), leukotoxin genes *luk*E/D [29], hemolysin genes (*hla*, *hlb* and *hld*) [29], and the *tst* gene encoding toxic shock syndrome toxin (TSST-1) [30].

Statistical analysis was performed in R (ver. 3.1.1; Vienna, Austria). Confidence intervals (95%) for individual groups of microorganisms and animal species and analysis of statistical significance were performed using Student’s T-test. The degree of variation in the frequency of appearance of potentially pathogenic bacteria with respect to the total number of isolates in each group of animals was tested by ANOVA. Statistical comparison of the potential epidemiological threat presented by individuals of different species was tested by Tukey’s HSD test. In both cases, the null and alternative hypotheses were based on average numbers of pathogenic bacteria in the analyzed groups of animal species.

## Results

The tests conducted on 421 animals made it possible to distinguish 328 isolates belonging to the genera *Salmonella*, *Staphylococcus*, *Listeria*, and *Yersinia* (Table 1).

**Table 1:** Species of bacteria isolated from free-living animals.

Species/serotype of bacteria	Number of positive isolates (%)			
	*Vulpes vulpes* (n = 286)	*Martes foina* (n = 65)	*Procyon lotor* (n = 70)	Total (n = 421)
*Salmonella enterica* subsp *enterica*	**7 (2.45)**	**6 (9.2)**	**2 (2.85)**	**15 (3.56)**
serotype Typhimurium,	3 (1.04)	3 (4.6)	1 (1.42)	7 (1.66)
Saintpaul	1 (0.35)	1 (1.5)		2 (0.47)
Infantis	1 (0.35)			1 (0.24)
Mbandaka,	1 (0.35)			1 (0.24)
Newport	1 (0.35)	1 (1.5)	1 (1.42)	3 (0.71)
Enteritidis		1 (1.5)		1 (0.24)
*Salmonella enterica* subsp *diarizonae*	**2 (0.69)**		**1 (1.42)**	**3 (0.71)**
*Salmonella enterica* subsp *houtenae*			**1 (1.42)**	**1 (0.24)**
***Salmonella* spp total**	**9 (3.15)**	**6 (9.23)**	**4 (5.7)**	**19 (4.51)**
*Staphylococcus aureus*	28 (9.8)	8 (12.3)	4 (5.71)	40 (9.5)
*Staphylococcus pseudintermedius*	172 (60.1)	10 (15.38)		182 (43.23)
*Staphylococcus delphini B*	2 (0.69)	7 (10.76)		9 (2.13)
*Staphylococcus delphini A*		2 (3.07)	21 (30)	23 (5.46)
***Staphylococcus* coagulase-positive strains total**	**202 (70.62)**	**27 (41.53)**	**25 (35.71)**	**254 (60.33)**
*Listeria monocytogenes*	13 (4.5)	4 (6.15)	3 (4.28)	20 (4.75)
*Listeria inocua*	5 (1.74)			5 (1.18)
*Listeria ivanovii*	1 (0.35)			1 (0.24)
*Listeria velshimerii*	3 (1.04)		2 (2.85)	5 (1.18)
***Listeria* spp total**	**22 (7.69)**	**4 (6.15)**	**5 (7.14)**	**31 (7.36)**
*Yersinia enterocolitica*	6 (2.09)	2 (3.07)	2 (2.85)	10 (2.37)
*Yersinia kristensenii*			1 (1.42)	1 (0.24)
*Yersinia frederiksenii*	10 (3.49)	3 (4.61)		13 (3.08)
***Yersinia* spp total**	**16 (5.59)**	**5 (7.69)**	**3 (4.28)**	**24 (5.7)**

The percentages of individual genera and groups of bacteria in the biota of the animals were varied and the samples isolated from each animal were positive for no more than three different bacterial species. The coagulase-positive *Staphylococcus* spp. were isolated most frequently (60.33%; CI: 27.8–35.53), while the presence of the other bacteria was detected in a smaller number of animals: *Listeria* spp. 7, 36%; (CI: 4.71–5.65), *Yersinia* spp. 5.7% (CI: 3.12–5.58), and *Salmonella* spp. 4.51% (CI: 1.31–6.2) (Table 1).

Of the total pool of 328 isolates, 90 isolates (21.34%) belonging to taxa posing the greatest potential threat to human health (considered mainly as foodborne pathogens) were selected for further analysis, including statistical analysis. This group included the following isolates: *Salmonella* spp. (n = 19), *Listeria monocytogenes* and *L*. *ivanovii* (n = 21), *Yersinia enterocolitica* (n = 10), and *Staphylococcus aureus* (n = 40). Irrespective of the animal species, *S*. *aureus* was isolated most frequently, accounting for 44.4% (CI: 42.23–46.57); *Listeria monocytogenes* and *L*. *ivanovii* (23.3%; CI: 22.83–23.77) and *Salmonella* spp. (21.11%; CI: 17.86–24.36) were isolated less frequently. *Y*. *enterocolitica* was the least frequent from the selected species of bacteria, accounting for 11.1% (CI: 10.71–11.5) of potentially pathogenic isolates. In general, one potentially pathogenic species was isolated from a single individual, except for 10 animals (6 foxes, 3 martens, and 1 raccoon), which were carriers of two different potentially pathogenic bacterial species at the same time. In most cases, the presence of *S*. *aureus* (80%; 8/10) was accompanied by *Salmonella* spp. (40%; 4/10), *L*. *monocytogenes* (10%; 1/10), or *Y*. *enterocolitica* (10%; 1/10). In one case, *L*. *monocytogenes* and *Y*. *enterococlitica* were isolated from a single individual (a fox) and, in another case, two different subspecies of *Salmonella* (*S*. subsp. *enterica* and *S*. subsp. *houtenae*) were isolated from a raccoon (Table 2).

**Table 2:** Distribution of potentially pathogenic bacteria in the free-living animal species studied.

Potentially pathogenic species/serotype	Host species		
	*Vulpes vulpes* (n = 286)	*Martes foina* (n = 65)	*Procyon lotor* (n = 70)
*S*. *enterica* subsp *enterica* serotype Typhimurium + *S*. *aureus*	1	1	
*Salmonella enterica* subsp. *enterica* serotype Saintpaul + *S*. *aureus*		1	
*S*. *enterica* subsp. *diarizonae* + *S*. *aureus*	1		
*L*. *monocytogenes* + *S*. *aureus*	2		
*L*. *monocytogenes* + *Y*. *enterocolitica*	1		
*Y*. *enterocolitica* + *S*. *aureus*	1	1	
*S*. *enterica* subsp. *enterica* serotype Typhimurium + *S*. *enterica* subsp *houtenae*			1
**Total mixed**	6	3	1
*Salmonella enterica* subsp *enterica*			
serotype Typhimurium,	2	2	
Saintpaul	1		
Infantis	1		
Mbandaka,	1		
Newport	1	1	1
Enteritidis	1		
*Salmonella enterica* spp *diarizonae*	1	1	
*Staphylococcus aureus*	23	5	4
*Listeria monocytogenes*	10	4	3
*Listeria ivanovii*	1		
*Yersinia enterocolitica*	4	1	2
**Total**	51 (12,11)	18 (4,27)	12 (2,85)

Taking into account the differences in the sizes of the samples from the different animal species (Student’s T-test; p < 0.05), we showed that on average for the entire group of animals (n = 421) the percentage of carriage of potentially pathogenic bacteria was 6.33%. The percentage of animals in which the presence of potentially pathogenic isolates was noted varied in the different groups; the highest percentage of individuals was noted in the group of foxes (n = 51; 12.11%; CI: 10.17–14.06), but it was much lower, i.e. 4.27% (CI: 2.93–5.15) and 2.85% (CI: 2.08–3.62), in the martens (n = 18) and raccoons (n = 12) (Table 2).

In the next stage of the study, the potential virulence of the isolates tested (*L*. *monocytogenes*, *S*. *aureus* and *Y*. *enterocolitica*) was analyzed on the basis of selected genes characteristic for a given bacterial species. The profiles of the virulence genes tested within the individual bacterial species were highly heterogeneous; 8 different profiles were found in the case of *L*. *monocytogenes* and 20 for *S*. *aureus* (Tables 3 and 4).

**Table 3:** Virulence profiles of *L*. *monocytogenes* isolated from free-living animals.

Number of *L*. *monocytogenes* isolates (n)%	Species host (n = 20)	Internalin genes	other genes detected
(7) 35	Fox(5),Marten (2)	*inl*A, *inl*C, *inl*J	*hly*A, *iap*, *plc*A, *prf*A, *act*A
(1)5	Fox(1)	*inl*A, *inl*C, *inl*J	*hly*A, *iap*, *plc*A, *prf*A
(2)10	Fox(2)	*inl*A, *inl*C, *inl*J	*hly*A, *iap*, *plc*A, *prf*A
(3) 15	Fox(1), Marten (1),Raccoon (1)	*inl*A, *inl*C, *inl*J	*hly*A, *iap*, *act*A
(2)10	Fox (1), Raccoon (1)	*inl*A, *inl*C, *inl*J	*hly*A, *iap*, *prf*A
(1)5	Fox (1)	*inl*A, *inl*C, *inl*J	*hly*A, *iap*
(1)5	Fox (1)	*inl*A, *inl*C	*hly*A, *iap*, *plc*A, *prf*A, *act*A
(3) 15	Fox (1),Marten (1)Raccoon (1)	*inl*A, *inl*C	*hly*A, *iap*, *prf*A, *act*A

**Table 4:** Virulence profiles of *S*. *aureus* isolated from free-living animals.

Number of *S*. *aureus* isolates (n) %	Species host (n = 40)	Enterotoxins genes detected	other toxins genes detected
(7)17.5	Fox(6),Marten (1)	*see*, *sei*	*hla*, *hld*,*hlb*,*luk*ED
(2) 5	Marten (1), Raccoon (1)	*see*, *sei*	*hla*, *hld*, *luk*ED
(1) 2.5	Marten (1)	[*seg*, *sei*, *sem*, *sen*, *seo*]	*tst*, *hla*, *hld*, *luk*ED
(2) 5	Fox(2)	[*seg*, *sei*, *sem*, *sen*, *seo*]	*hla*, *hld*, *luk*ED
(4) 10	Marten (2), Fox (2)	*sea*, *seb*, *seg*, *sed*	*hla*, *hld*, *luk*ED
(1) 2.5	Fox (1)	[*seg*, *sei*, *sem*, *sen*, *seo*]*sec*	*hla*, *hld*,*hlb*, *luk*ED
(1) 2.5	Fox (1)	[*sem*, *sen*, *sei*]	*tst*,*hla*, *hld*, *luk*ED
(1) 2.5	Fox (1)	*sea*, *see*, *sed*	*hla*, *hld*, *luk*ED
(1) 2.5	Fox (1)	*sea*, *see*	*hla*, *hld*, *hlb*, *luk*ED
(1) 2.5	Fox (1)	*sed*, *sej*	*hla*, *hld*, *luk*ED
(2) 5	Fox (2)	*seh*	*hla*, *hld*,*hlb*, *luk*ED
(2) 5	Fox (1), Marten (1)	*seh*	*hla*, *hld*
(1) 2.5	Fox (1)	*sei*	*hla*, *hld*, *luk*ED
(1) 2.5	Fox (1)	*sej*	*hla*, *hld*, *luk*ED
(1) 2.5	Raccoon (1)	*see*	*hla*, *hld*, *luk*ED
(1) 2.5	Raccoon (1)	*see*	*hla*, *hld*
(2) 5	Fox (2)	*sec*	*hla*, *hld*, *luk*ED
(2) 5	Fox (2)		*hla*, *hld*, *luk*ED
(5) 12.5	Fox (3), Marten (2)		*hla*, *hld*
(2) 5	Fox (1), Raccoon (1)		

The distribution of the particular virulence genes in the pool of 20 isolates of *L*. *monocytogenes* was varied, both quantitatively and qualitatively; the presence of all genes tested (*inl*A, *inl*C, *inl*J, *hly*A, *iap*, *plc*A, *prf*A and *act*A) was noted in 35% of the isolates, while the number of genes in the other isolates ranged from 5 to 7. The *inl*A, *inl*C, *hly*A and *iap* genes were present in all the tested isolates of *L*. *monocytogenes*, while genes *inl*J, *plc*A, *prf*A and *act*A occurred with varying frequency: 80% (CI: 78.23–81.77), 55% (CI: 53.31–59.69), 70% (CI: 68.65–71.35), and 80% (CI: 78.51–81.49), respectively. The virulence profiles of the 40 *S*. *aureus* isolates were determined on the basis of the presence of toxin-encoding genes (Table 4).

Most of the isolates (95%; CI: 93.41–96.59) contained hemolysin-encoding genes *hla* and *hld*, whereas gene *hlb* was noted in only 32.5% (CI: 30.27–34.73) of the isolates. The *luk*ED gene encoding bicomponent leucotoxins was detected in 70% (CI: 68.29–71.71) of *S*. *aureus* isolates. Two strains, from a fox and a marten, showed the presence of the gene encoding TSST-1. Genes encoding enterotoxins were detected in 77.5% (CI: 79.88–79.12) of the isolates, including five (12.5%) with *egc-*like variants (Table 4). No genes encoding leukocidins (*luk*S/F-PV and *luk*M) were observed in any of the *S*. *aureus* isolates.

In the case of *Y*. *enterocolitica*, the presence of the *ail* gene was found in 60% of isolates (Table 5); only isolates from martens were all positive for this gene.

**Table 5:** Number and prevalence of pathogenic *Yersinia enterocolitica* in free-living animals.

Animal species (positive isolation of *Y*. *enterocolitica*)	positive PCR amplification results (%)	
	16S rRNA gene (330bp)	*ail* gene (425bp)
*Vulpes vulpes* (n = 6)	6 (100)	3 (50)
*Martes foina* (n = 2)	2 (100)	2 (100)
*Procyon lotor* (n = 2)	2 (100)	1 (50)
Total (n = 10)	10 (100)	6 (60)

## Discussion

Free-living animals, particularly predatory species, are a significant link in the epidemiological chain of most zoonoses, mainly as a reservoir for transmission of zoonotic agents to humans and domestic animals [1]. In Poland, there has been little comprehensive research of this issue; more extensive research has been conducted only in a deer population [31]. Our analysis focused on evaluating the degree of carriage of potentially pathogenic bacteria of the genera *Salmonella*, *Yersinia*, and *Listeria* as well as coagulase–positive *Staphylococcus* in three most common carnivorous animal species in Poland.

Foxes, which constituted the largest group of animals in the present study, belong to species with a strong focus on synanthropization. Due to the fact that intensive vaccination against rabies used in the recent years led to a significant increase in their population, the animals began to change their habitat and move to regions populated by humans more densely (high availability of food resources). A significant percentage of the samples (40%) were taken from foxes suspected of having rabies, and the animals came mainly from urban and suburban areas, where the human population density ranged from 200 to 2000 persons/km^2^. The other samples came from foxes culled as part of monitoring tests, and these animals were obtained from rural areas (with a density of 20–100 persons/km^2^) and uninhabited or sparsely populated areas with a density of less than 20 persons/km^2^.

In the case of the marten, all animals were from both rural and urban areas inhabited by humans (including urban green areas such as parks and gardens). This is not a surprising phenomenon, because the beech marten is closely related to the human environment, nesting in places such as farm buildings and houses. Although raccoons have been caught in the National Park, it should be emphasized that this area is directly adjacent to the urban area (e.g. Kostrzyn city). Analysis of the direct impact of this species of animals on humans should take into account the fact that raccoons, as synanthropic animals, strongly penetrate the direct human environment [32,33] and have a very high potential of migration and a very dynamic growth of the population size [34].

The presence of these animals in the human environments is not only connected with the possibility of transferring microorganisms by direct contact, both in urban and rural human populations as well as in groups of increased risk (forest guards, veterinary staff, hunters, wildlife officers), but also by indirect contamination of the environment by feces. Pathogens found in feces can contaminate water and soil, and in this way they can contaminate the food of plant origin (e.g. raw fruit and vegetables) [35]. According to the report of the European Commission of Health and Consumer Protection [36], all bacterial species examined in this study as potential foodborne pathogens can contaminate agricultural produce and have a capacity to cause outbreaks.

The considerable percentage of the positive isolations of *Salmonella* spp. (4.51%) was generally consistent with the results of studies carried out in similar animal populations in Italy, Spain, Norway, the USA, and Japan, which suggests the need for international monitoring of free-living animals in this regard. In the case of detection of *Salmonella* carriage, continual monitoring is currently only required for farm animals [37], while research on other groups of animals is fragmentary; in Poland, it has concerned mainly living birds and cold-blooded vertebrates [14, 38].

The degree of *Salmonella* spp. carriage is varied and depends in part on the host species (host-restricted, host-adapted, and ubiquitous). In the pool of the animals analyzed, the highest percentage of positive isolations was obtained in the martens (9.2%). Similar results were noted in Italy [39], whereas in Spain [40] no positive isolations of *Salmonella* were found in this species. In the case of foxes, the frequency of *Salmonella* isolation was lower than that reported by other authors (5.2% to 12.5%) [39, 40, 41], which may be linked to a certain seasonality of *Salmonella* occurrence in foxes [42] and to climatic determinants influencing the survival and distribution of these bacteria in the environment [43].

As a reservoir of *Salmonella* spp., the raccoon ranked between the two species discussed above; the percentage of positive isolates was 5.7%, which was consistent with other research [32, 44]. It is interesting that two subspecies of *Salmonella*, *S*. *enterica* subsp. *enterica* and *S*. *enterica* subsp. *houtenae*, were both present in the biota of the digestive tract of a single individual. A similar phenomenon was observed in a previous study [38], which clearly suggests the need to include more than one colony isolated from a given sample in the isolation and identification of *Salmonella*. Among the six *S*. *enterica* subsp. *enterica* serotypes isolated in our study, four (*S*. Typhimurium, *S*. Infantis, *S*. Newport, and *S*. Enteritidis) are included among the 10 serotypes of non-typhoidal *Salmonella*, which are most often responsible for infections in humans in Europe, including Poland [45]. In Poland, *Salmonella* Typhimurium is the second most frequently isolated serotype in cases of salmonellosis in humans [46] and carriage in farm animals [47] and free-living birds [14]; as shown in the present study, it is also the most frequently isolated serotype among *S*. *enterica* subsp. *enterica* in free-living carnivorous animals.

Due to the fact that *Salmonella* is a multi-host pathogen and can persist in the environment for long periods, there are only a few cases documenting a direct relationship between the wildlife source and the occurrence of salmonellosis in humans [48, 49] or livestock [5], similar to the documented cases of direct contamination of fresh produce by free-living animals [49].

However, the possibility of *Salmonella* circulation between humans and free-living animals (or livestock as an indirect link) is very complex, and in many stages both direct and indirect contact can lead to transmission of *Salmonella* [35]. Likewise, the source of contamination of the free-living animals analyzed in the present study is not exactly known. Probably, the carriage may be linked to the type of animal diet covering e.g. small rodents or free-living birds. Many authors have shown a significant role of mice, rats, hedgehogs, amphibians, reptiles, and free-living birds as a reservoir of *Salmonella* [14, 50, 51].

Another group of bacteria with considerable zoonotic potential that was isolated from carnivorous species of free-living animals was *Yersinia* spp. (5.7%). According to the European Centre for Disease Prevention and Control [37], *Y*. *enterocolitica* is the cause of 96.5% of cases of yersiniosis in humans, while the second pathogenic species belonging to this genus, *Y*. *pseudotuberculosis*, is responsible for only 1.9% of infections. The main route of infection is foodborne transmission. Infection may also occasionally result from indirect contact with infected animals, including free-living animals (e.g. via water, soil, and vegetables contaminated by such animals) [52]. Most studies on the occurrence of *Yersinia* spp. among free-living animals have concerned wild boars as a potential (analogously to the pig) reservoir of *Y*. *enterocolitica* [53, 54]. Other species of free-living animals have rarely been studied and these have usually been small groups of animals [55, 56]. An exception is a study by Lee et al. [32] carried out in a raccoon population, in which *Y*. *pseudotuberculosis* was found to be the dominant isolated species. In our study, the degree of carriage of *Yersinia* spp., irrespective of the host species (fox, marten, or raccoon), was similar, i.e. 5.7%, and 41.6% of all *Yersinia* isolates were *Y*. *enterocolitica*. The other species, *Y*. *kristensenii* and *Y*. *frederiksenii*, accounted for 4.2% and 54.1% of all *Yersinia* isolates. As their role in the pathogenesis of infections has not been established [52], they were not included in further analyses. Detection of the presence of the *ail* gene, responsible for attachment and invasion, in 60% of the *Y*. *enterocolitica* isolates confirms the potential health risk for humans and animals posed by the presence of these bacteria in the digestive tract of these carnivorous animal species. Direct contact with infected (carrier) individuals or the environment contaminated these bacteria may be an important element of the epidemiological chain, not only in the case of yersiniosis but also listeriosis [57, 58].

Among all species of *Listeria* spp., mainly *Listeria monocytogenes* and *L*. *ivanovii* are regarded as potentially pathogenic, with the latter species primarily associated with infections in animals [25, 59, 60]. Among all the strains of *Listeria* isolated in the present study, *L*. *monocytogenes* comprised the largest group (20/31), and the overall percentage of isolation was similar to results obtained by other authors in wildlife animals [61, 62]. *L*. *monocytogenes* is pathogenic for human population groups, but poses the greatest threat to infants, pregnant women, and elderly immunosuppressed individuals [63]. Pathogenic strains must be differentiated from non-pathogenic ones, particularly in the case of isolation from the environment. Virulence and pathogenicity factors of *L*. *monocytogenes* strains, such as internalin, listeriolysin O, actin, phosphatidylinositol phospholipase C, IAP proteins associated with invasiveness, and virulence regulators have been found both among strains isolated from disease cases, such as miscarriage in women [64], and in environmental strains [25]. Markers identifying strains as virulent include the presence of the gene encoding internalin lmo2821 (*inl*J), which is a marker differentiating virulent strains causing mouse mortality from non-virulent strains [65]. This gene was found in 80% of *L*. *monocytogenes* isolates from the animals in our study. The gene encoding phosphatidylinositol phospholipase C (PI-PLC), which has been detected only in pathogenic strains of *L*. *monocytogenes* and *L*. *ivanovii* [64], seems to be equally significant. In the present study, the gene encoding PI-PLC was present in 55% of *L*. *monocytogenes* isolates—only those from foxes and martens. The other virulence genes identified (inl*A*, inl*C*, prf*A*, act*A*, hly*A*, and *iap*) occurred in varying numbers and configurations in all strains from which *L*. *monocytogenes* was isolated (8 separate profiles), with the full panel of virulence genes noted in 7 of the 20 isolates. These results indicate that these isolates possess all the properties of a virulent strain and are very likely to pose a direct threat to the health of humans and animals (including livestock).

The most frequently isolated group of bacteria in the pool of the animals studied was coagulase-positive *Staphylococcus* (60.33%). These are commonly occurring bacteria colonizing the bodies of humans and animals, and their interaction with the host can be either commensal or opportunistic [66]. *S*. *aureus* is responsible for most infections induced by this group of bacteria in humans [67] and constitutes one of the important causes of foodborne diseases [68, 69, 70]. Among all coagulase-positive *Staphylococcus* species, *S*. *aureus* is the main described cause of staphylococcal foodborne outbreaks [70]. Other species, especially belonging to the *Staphylococcus intermedius* group, have low enterotoxigenic potential and only occasionally induce cases of disease [71]. *S*. *aureus* compared with other genera, i.e. *Salmonella*, *Campylobacter*, or *Shigella*, seems to account for a small percentage among documented bacterial causes of foodborne diseases reported to public health agencies, but the true number of cases of *S*. *aureus* foodborne disease may be much higher for various reasons, most often associated with a lack of laboratory confirmation of the presence of SEs in clinical stool samples [68]. Therefore, *S*. *aureus* should also be included in analysis of the presence of potential pathogens in free-living animals. A broad and diverse panel of factors allowing *S*. *aureus* to adapt particularly well to the human body [72] by colonizing and/or infecting it may also be active in the case of other mammalian hosts [73]. In our study, *S*. *aureus* was isolated at a similar level from all the animal species and, as in studies by other authors [74, 75, 76], it was not a dominant species among coagulase-positive *Staphylococcus* spp. The vast majority of isolates (38/40) had genes encoding virulence factors occurring in varying configurations (number/type of gene). Among the 40 *S*. *aureus* isolated in this study, 31 were characterized by the presence of genes encoding at least one type of enterotoxin (SEs). Enterotoxins belonging to the family of pyrogenic toxin superantigens (PTSAg) have been linked to food poisoning and, as superantigens (SAg), can lead to potentially lethal toxic shock syndrome [70]. In five strains, there were also enterotoxin gene cluster (*egc*) or *egc-*like variants, which were detected in over half of all *S*. *aureus* strains, both commensal and invasive [77]. Novrousian et al. [77] demonstrated that the presence of an *egc* locus can facilitate colonization by *S*. *aureus* of mucosal surfaces and increase weight loss in an experimental model of *S*. *aureus* infection. The *hla* and *hld* genes encoding hemolysins α and δ, leukotoxin LukE-LukD (30/40), and hemolysin β (11/40) were noted most frequently. The virulence factors such as leucotoxins have been demonstrated to kill human neutrophils, but only LukE-LukD leucotoxin exhibited lytic activity against red blood cells [78]. Genes encoding leukotoxin LukE-LukD were present in most *S*. *aureus* strains isolated from cases of mastitis in cows [79] and in blood isolated from humans with *S*. *aureus* bacteremia [80]. Hemolysins, especially α-hemolysin, are important virulence factors in several animal models of infection, including pneumonia and mastitis [81] The presence of the *hla*, *hld*, and *hlb* genes and the enterotoxin gene cluster has also been shown in *S*. *aureus* strains from cases of mastitis in cows in Poland [82, 83].

The presence of the *tst* gene encoding toxic shock syndrome toxin-1 (TSST-1) in two (5%) strains isolated from a fox and a marten in the present study is noteworthy. TSST-1 has also properties of a superantigen and its presence is connected with toxic shock syndrome (TSS), a rare but important cause of morbidity and mortality [84]. The presence of this toxin is also related to the etiology of many other diseases or syndromes in humans [73]. The *tst* gene has not been detected so far in isolates from companion animals and livestock (dogs, cats, pigs, or chickens) in Poland [82], but it has been present in isolates from neonatal intensive care units in Polish hospitals, usually in combination with the enterotoxin gene cluster (*egc*) and *egc-*like variants [85]. This phenomenon may indicate that the presence of *S*. *aureus tst* gene positive strains in free-living animals may be associated rather with their exposure to direct human sources (e.g. sewage, waste from hospitals), as in the case of our previous studies on isolation of a community-acquired methicillin-resistant *S*. *aureus* (CA-MRSA) strain from a marten [86].

In the case of free-living animals, the level of carriage of specific microbes is most often analyzed [41, 53, 86, 87, 88]. Confirmed cases of illness are noted less frequently (salmonellosis, listeriosis, yersiniosis, or local and generalized *S*. *aureus* infection) [33, 48, 89, 90] and the primary source of infection/carriage of these animals is analyzed extremely rarely [91]. Due to their varied diet, carnivorous animals may constitute an accumulation vector by eating small mammals, birds, and even insects that may be carriers of pathogens and transmit them in contact with humans or domestic animals. Most animals (foxes and martens), in the present study came from areas that include provinces with the largest number of farms producing organic food, including farm animal. One of the requirements for organic farming is to keep farm animals with outdoor access, which greatly increases the possibility of contact of free-living animals with farm animals and, thus, the potential transmission of pathogens [35].

Apart from the transfer from free-living animals to humans, a reverse phenomenon can occur. As a result of increasing anthropogenic changes in the environment leading to greater interpenetration of communities of humans and free-living animals, exposure of animals to pathogens that are present in the human environment or livestock (waste, manure from farm animals) can occur. Messenger et al. [92] showed that 50% of all recorded infections in animals related to reverse zoonosis apply to free living animals, where direct contact was suggested as the most frequent transmission route.

The present study showed that predatory species of free-living animals can be a significant reservoir of potentially pathogenic bacteria. Among the species analyzed (the red fox, beech marten, and raccoon), the fox may pose the greatest threat, due to its continually growing population size and occurrence all over Poland, as well as the statistically significantly higher frequency of isolation of bacteria that are potentially pathogenic for humans and other animals. To analyze the importance and participation of free-living carnivorous animals in the chain of transmission and spread of the microorganisms analyzed in the present study, additional research should be carried out covering the lower floors of the food chain (small mammals and birds, amphibians, reptiles, insects) and the natural habitat of carnivorous animals (water, soil, plants). Moreover, given the virulence potential of the isolated strains (the presence of virulence genes), it is clear that expanding epidemiological monitoring to include free-living animals must be considered.
